# Journal of Pharmacy

**Published:** 1836

**Authors:** 


### Art. VI. Journal of Pharmacy.

*The American Journal of Pharmacy, published by authority of the Philadelphia College of Pharmacy.* Edited by R. E. Griffith, M. D.

We have before us the third number of the seventh volume, for Oct. 1835, of this useful work. For not having mentioned it before, we owe an apology, both to our readers and to the College, under whose auspices it is published. Its contents are original and selected; the former predominating, and consisting in a great degree, of papers from the current periodicals of Europe and America. Nevertheless they are interesting, because it is useful to have them collected and arranged in connexion with each other. On this account alone, the Journal deserves a far greater patronage, than it has received in the West, where indeed it is almost unknown. The value of this work is, however, increased by the communications which make up its original department, the most important of which, in the number before us, are from the pen of its talented Editor. We do not find in these papers, however, any thing specially deserving of analysis.

Of the selections, the first is a paper from the American Journal *of the Medical Sciences*, (which not having mentioned at the time of its publication, we shall notice here,) by Dr. Mettauer, of Virginia. It is on the use in Epilepsy, of the *Crusta Genu Equinæ*, hangers, or knee scab of the horse.

"The substance designated by the several appellations at the head of this article, is furnished by the horse; four oval surfaces, situated on the inner aspects of the extremities, near the knees, are the parts of the animal from which it is obtained. The secretion is poured out so gradually, and in such small quantities at a time, as not to be observed in its fluid, or even semi-fluid states. The crust is of variable color, as well as density; its exterior is always of a lighter appearance, and harder than the interior, which is dark and soft; it is of a lamellated and fibrous texture, and when broken, resembles dark, soft horn; its odor is very penetrating, diffusible, and peculiar; it is deciduous, and separates gradually two or three times during the year; when prematurely or forcibly removed, the surface from which it is taken, sometimes bleeds a little, inflames, and becomes tender and sore.

"In collecting the crust for medical purposes, it is necessary to attend carefully to its loosening tendency from time to time, or it may fall off and be lost. It may be made to separate a little sooner by gentle solicitation, and occasionally by firm compression with a bandage.— This should be suffered to remain on after the period of disquamation is near at hand, to prevent the accidental loss of the crust. After it is obtained, it should always be dried a short time in the shade, and then it may be kept for use in a close jar, to prevent, so far as possible, the escape of its volatile properties.

"Two forms for administration are only used—the powder and tincture. When the powder is to be used, it should always be freshly prepared, either by pounding and rubbing the dry crust in a mortar, or by grating it with a common nut-meg grater: this last process will be found, (generally,) most convenient, as it enables the practitioner to reduce it, at once, to a very fine and equable powder, even if the crust is imperfectly dried.

"The tincture is prepared by simply digesting the broken or powdered crust in diluted alcohol, or common brandy, exposed to a gentle heat for eight or ten days in the proportion of one part of the former to four of the latter.

"The doses of the powder vary from two to twenty grains; it may be given diffused in any liquid which the patient fancies. With young patients it is safest to begin with the minimum, and increase very gradually to the maximum doses. Should the disease yield before the largest doses are reached, no further augmentation need be made. When the tincture is employed, from ʒss. to ℥iss. are its extreme doses. Diluted with water and sugar, it may be given with very little difficulty to the youngest subjects, as it is tasteless, and in a great measure inodorous. In this form also, the doses should be very gradually increased, to prevent, as far as possible, the danger of exciting the system too much, which might result from the menstruum, should the doses be suddenly augmented."

As the title of this paper implies the *Crusta*, is to be employed in Epilepsy. It should always be preceeded by mild purgation, and sometimes by blood letting. When the temperament of the patient is sanguine, the medicine should be given in substance.

"With young subjects from six to eight years of age, two grains will, in a majority of cases, constitute the commencing dose. We have never used it with patients younger than six years, or older than thirty. Older patients, say from eight to twelve, or fifteen years of age, will bear four or five grains, or even larger doses in the commencement, and with such it may be more suddenly increased to the maximum doses, without gastric disturbances. The remedy rarely offends the stomach when the foregoing precautions are properly attended to; on the contrary, it seems rather to compose and tranquilize this organ. Three doses, in a majority of cases, are as many as will be required in the twenty-four hours. Should cases occur marked by convulsions of unusual violence, with frequent paroxysms, it may be given oftener. From many trials with this article, it has not been perceived that there is much diversity of effect when employed in large or medium doses with young subjects."

The tincture is to be preferred in those who are but little inclined to a phlogistic diathesis, and from half a drachm to an ounce, and a half may be given three or four times a day.

"In obstinate cases the crust should be continued for more than a year before it is to be discarded, or the case abandoned as incurable; both forms should always be employed and used alternately.

"The crust in form of tincture is also a valuable nervine and antispasmodic in hysteric convulsions, and indeed in hysteria generally. In that variety, connected with or proceeding from uterine irregularities incident to sterile married (or unmarried,) females, it will be particularly serviceable; with such the paroxysms most strikingly resemble epilepsy."

The object of this paper being chiefly to commend to the notice of our readers, the Journal of Pharmacy, we shall content ourselves with placing before them a single additional extract.

"Mr. C. Texier, who is at present exploring Asia Minor, has transmitted from Constantinople to the Academy of Sciences of Paris, the following details respecting the cultivation of the poppy and the preparation of opium.

The seeds are sold at Kara-Hissar by measure of 60 ocques at 20 paras, the ocque, that is 30 piastres, (or about a dollar and a half.) The ocque of Constantinople is equal to about 2¾ pounds. They begin to work the earth in December by means of a hoe, or sometimes with a plough. The furrows are sufficiently large to permit persons to pass without damaging the stems of the poppies, which are planted in beds of three feet and a half wide. The seeds are sown broad cast but very thin. One ocque is sufficient to sow 1600 square metres. A few days after the flowers have fallen, the heads are slit horizontally, taking care that the cut does not penetrate to the interior. A white milky juice exudes, which is left for twenty-four hours, and then scraped off with large, dull knives. Each head furnishes but a few grains of opium. The drug is sophisticated by portions of the epidermis being mixed with it, thus increasing the weight about a twelfth. The opium is now in the form of a sticky granular jelly. It is placed in small earthen vessels and pounded, the operator spitting in it from time to time. When the peasants are asked why they do not use water instead of saliva, they reply that water would injure it, the opium is then wrapped in dry leaves, and is fit for sale. The seed is not injured by cutting the heads.

" The quality and abundance of the crop are favored by the absence of heavy rains during May and June, a few days rain being sufficient to cause a great loss.

"On the above being read, M. Guibourt stated that in his opinion this mode of collecting and preparing opium, was peculiar to the spots visited by M. Texier, but that it is not probable that the opium of commerce is thus manufactured; as it is not noticed by other travelers, and also that an inspection of good opiums of commerce, show that they have not undergone such manipulations.

"M. Guibourt has already proved (*Dict. de Med. et de Chirur. prat.* art. Opium,) that it is erroneous to suppose that we do not possess the true opium of the ancients, or the product of an incision of poppy heads; and to say that we have only the *meconium* or the product of the expression or decoction of the plant. M. Guibourt is satisfied that the Smyrna and even good Constantinople opium, (grown in part of Natolia) are the product of incisions of the poppy head, and adduces in proof, that by carefully tearing these opiums and observing the fracture with a magnifying glass, they appear to be formed of small tears or drops, agglutinated together. It is evident that they have undergone no other preparation than that of being formed into cakes, and when sufficiently dried, each enveloped in a leaf of the plant, and in the Smyrna opium also with seeds of a *Rumex*, which however does prevent the cakes, in many instances, sticking together and becoming united.

"This Smyrna opium exactly agrees with the description of Belon: 'The best of opium, says he, is very bitter, hot to the taste so as to burn the mouth. It is of a yellow color, approaching that of the skin of a lion, the masses are composed of a number of small grains of different shades of color. For in collecting the said opium, the grains obtained from the different poppy heads are united together."

Oliver does not speak of any other preparation of opium, and the concurrence of these two travelers, joined to the physical characters of the smyrna opium, does not furnish a doubt of the mode in which it is manufactured.

But another method of preparing it does exist; this is mentioned by Dioscorides and Kœmpfer. According to the former, the juice collected from the capsules, for two successive days, is mixed together in a mortar; according to the latter, 'it is moistened with a *little wa-*

*ter*, so as to soften it, when it is well worked in a wooded bowl, with a wooden spatula, till it acquires the consistence, color and tenacity of malaxated pitch. When it has been thus worked, it is several times stretched out and rolled with the hands, and then made into cylinders.'

The opium prepared by either of these processes will not present the drops or tears so distinct in the Natolia, but will resemble the uniform texture of the Egyptian and Persian. M. Guibourt has specimens of the Persian opium given to him by Mr. Morrison, of London, which has all the characters spoken of by Kœmpfer. It is in cylindrical pieces, which have sometimes become square from their pressure on each other; they are about four inches and a half long, and about five or six lines thick, wrapped in satin paper and tied with a cotton thread. Each stick weighs about 20 grammes; internally they are uniform, reddish, presenting some appearance of agglutinated tears, when viewed with a magnifying glass, but of a smaller size than those of the Smyrna opium. It has a virous smell, mingled with mustiness, which also characterizes the Egyptian variety, prepared in the same manner.—*Jour. de Pharm.*

